# Doxorubicin‐induced heart failure in cancer patients: A cohort study based on the Korean National Health Insurance Database

**DOI:** 10.1002/cam4.1886

**Published:** 2018-11-19

**Authors:** Young Ae Kim, Hyunsoon Cho, Nayoung Lee, So‐Youn Jung, Sung Hoon Sim, In Hae Park, Sunmi Lee, Eun Sook Lee, Hak Jin Kim

**Affiliations:** ^1^ National Cancer Control Institute National Cancer Center Goyang‐si Korea; ^2^ Department of Cancer Control and Population Health, Graduate School of Cancer Science and Policy National Cancer Center Goyang‐si Korea; ^3^ Center for Breast Cancer National Cancer Center Goyang‐si Korea; ^4^ Health Insurance Policy Research Institute National Health Insurance Service Gangwon‐do Korea; ^5^ Department of Cardiology, Center for Clinical Specialty National Cancer Center Goyang‐si Korea

**Keywords:** doxorubicin, heart failure, incidence, risk factor, survival

## Abstract

**Background:**

Doxorubicin is a typical anticancer drug that causes cardiomyopathy and heart failure (HF). The aim of our study was to investigate incidence, risk factors for doxorubicin‐induced HF in Korean cancer patients and their survival rate, utilizing a nationwide population‐based cohort.

**Methods:**

We analyzed 58 541 cancer patients who received doxorubicin between 2003 and 2010. Descriptive analysis was performed in patients with breast cancer, hematologic malignancy, gynecological malignancy, and sarcoma. Risk factors associated with doxorubicin‐induced HF were investigated using a Cox proportional hazards model. The survival rate of doxorubicin‐induced HF patients was compared with that of patients without doxorubicin‐induced HF.

**Results:**

A total of 2324 (4%) were diagnosed with doxorubicin‐induced HF. In patients with breast cancer, predictive risk factors for doxorubicin‐induced HF included age over 65 years [hazard ratio (HR) 1.34, 95% confidence interval (CI) 1.05‐1.72], hypertension [HR 2.45 (2.12‐ 2.84)], diabetes mellitus [HR 1.26 (1.05‐1.51)], coronary artery disease [HR 2.08 (1.63‐2.66)], advanced stage [HR 1.31 (1.13‐1.50)], and trastuzumab administration [HR 2.94 (2.54‐3.40)]. In patients with hematologic malignancy, predictive risk factors included age over 65 years [HR 1.75 (1.49‐2.07)], hypertension [HR 1.62 (1.37‐1.92)], and coronary artery disease [HR 2.28 (1.80‐2.89)]. Five‐year survival rates of patients with doxorubicin‐induced HF were significantly lower relative to those of patients without HF in breast cancer and hematologic malignancy: 80% vs 84% and 69% vs 75%, respectively (*P* < 0.001).

**Conclusions:**

In cancer patients treated with doxorubicin, management of risk factors, early detection, and treatment for doxorubicin‐induced HF might be critical for patient survival.

## 1 INTRODUCTION

Cancer treatment has markedly improved due to advances in cancer screening and the development of anticancer strategies, while the number of cancer survivors increases every year.^1^, ^2^ However, chemotherapeutic agents can also induce complications in cancer patients that delay or interrupt treatment. Such chemotherapy‐induced complications may decrease quality of life and increase mortality in cancer survivors.^3^, ^4^ One common type of chemotherapy‐induced complication is heart failure (HF). To date, a typical chemotherapeutic agent that causes HF is known as anthracyclines.^5^, ^6^, ^7^ They are antitumor antibiotics that are used to treat breast cancer, lymphoma, leukemia, sarcoma, and other solid tumors.^8^, ^9^ Anthracycline‐induced cardiotoxicity is now understood to be a continuum that begins with subclinical myocardial injury and develops into asymptomatic left ventricular (LV) dysfunction and subsequent symptomatic HF.^10^, ^11^ Therefore, early diagnosis and treatment are critical for reversible normalization of doxorubicin‐induced HF.

The most common anthracycline is doxorubicin. It is also the prototype for type 1 chemotherapy‐induced cardiotoxicity characterized by myocardial cell death, rendering dose‐dependent, and irreversible injury.^11^ Importantly, the risk of doxorubicin‐induced HF increases with cumulative dose. The maximal standard cumulative dose for doxorubicin is 400‐450 mg/m^2^, with an HF prevalence of approximately 5%.^12^ Several risk factors are associated with anthracycline‐induced HF including female, age (>65 years, <18 years), radiation therapy involving the heart, concomitant cardiotoxic chemotherapeutics, and preexisting cardiovascular risk factors such as smoking, hypertension, diabetes mellitus, and coronary artery disease.^13^ The 5‐year survival rate of doxorubicin‐induced HF is estimated to be <50%, with a worse prognosis than idiopathic or ischemic cardiomyopathy.^14^ However, these results have been reported mainly in Western cancer patients, while few studies involving Asian cancer patients have confirmed the incidence, risk factors, and survival rate of doxorubicin‐induced HF. Thus, the aim of our study was to investigate these variables for doxorubicin‐induced HF in cancer patients in a nationwide cohort.

## 2 MATERIALS AND METHODS

### 2.1 Data sources

The study protocol was approved by the Institutional Review Board of the National Cancer Center in Korea (NCC2016‐0142). Our study included cancer incidence data from the Korean Central Cancer Registry (KCCR). Briefly, patients diagnosed with cancer at a hospital are entered into the KCCR, and their data are used to calculate cancer registration statistics such as cancer incidence, survival rate, and prevalence. These data cover approximately 95% of all newly diagnosed malignant tumor cases in Korea.^15^ The National Health Insurance Service of Korea (NHIS) covers health insurance claims and health screening data from 2002 to 2012. The present study targeted cancer patients listed from 2003 to 2010. The final subjects were selected from 1 225 218 patients registered in the KCCR and linked to their NHIS data including general health screening, health care utilization, and insurance usage.

### 2.2 Study population

The present study used cancer incidence data registered with KCCR (2003‐2010), health insurance claims and health care utilization data from NHIS (2003‐2010), and cause of death data from Statistics Korea (2003‐2011).

**Figure 1:**
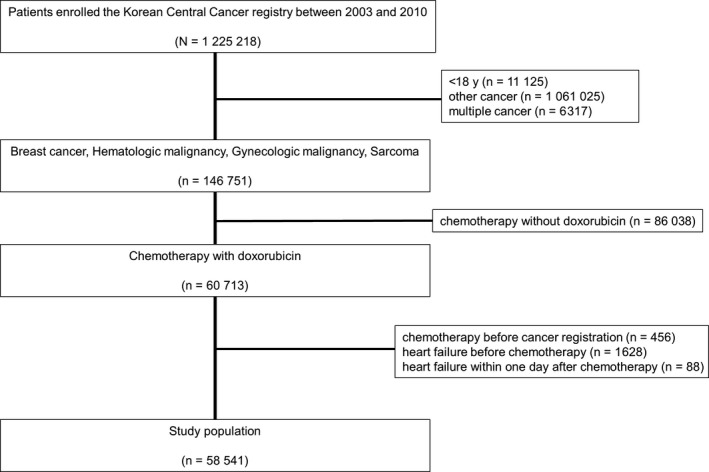
Study flow diagram

Among the 1 225 218 patients diagnosed with cancer and registered with the KCCR between 2003 and 2010, 146 751 patients were selected who were ≥18 years and diagnosed with any of the following: breast cancer, hematologic malignancy (non‐Hodgkin lymphoma, Hodgkin lymphoma, acute lymphoblastic leukemia, and acute myelogenous leukemia), gynecologic malignancy (ovarian cancer, endometrial cancer, and uterine sarcoma), and sarcoma (soft tissue sarcoma and osteosarcoma). Patients with multiple primary cancers were excluded. Among the 146 751 patients with a single primary cancer, 58 541 patients were selected who underwent chemotherapy after cancer diagnosis but did not show HF prior to or within one day of chemotherapy administration (Figure 1).

### 2.3 Definitions

We defined HF according to disease codes: International Statistical Classification of Diseases and Related Health Problems 10th Revision (ICD‐10). Patients were assigned to the HR group if they experienced cardiomyopathy due to drugs and other external agents (I42.7), cardiomyopathy (I42), other cardiomyopathies (I42.8), unspecified cardiomyopathy (I42.9), HF (I50), congestive HF (I50.0), congestive HF with systolic dysfunction (I50.04), other and unspecified congestive HF (I50.08), LV failure (I50.1), and unspecified HF (I50.9). All others were assigned to non‐HF group. Other diseases were classified and defined according to ICD‐10: I10, I11, I12, and I13 for hypertension; E10, E11, E13, and E14 for diabetes mellitus; E78 for dyslipidemia; and I20, I21, I22, I23, I24, and I25 for coronary artery disease.

The subjects were divided by cancer disease codes: C50 for breast cancer; C81, C82, C83, C85, C86, C91, and C92 for hematologic malignancy; C54, C55, and C56 for gynecologic malignancy; and C40, C41, and C49 for sarcoma. For each cancer type, subjects were divided into HF and non‐HF groups. The general health screening data collected closest to the date of cancer onset were used to obtain their body mass index (BMI), frequency of smoking, smoking cessation, and nonsmoking status.

### 2.4 Statistical analysis

Descriptive analysis was performed on age, gender, BMI, smoking, hypertension, diabetes mellitus, dyslipidemia, coronary artery disease, Surveillance, Epidemiology, and End Results (SEER) summary stage, radiation therapy, and number of cycles of doxorubicin in patients with breast cancer, hematologic malignancy, gynecologic malignancy, and sarcoma. In breast cancer patients only, frequency analysis was performed on trastuzumab after doxorubicin administration. Chi‐square tests and Student's *t* tests were performed to evaluate statistically significant differences between HF and non‐HF groups.

To investigate the risk factors that affect HF patients and survivors, a Cox proportional hazards model was used to estimate hazard ratios (HRs). Age, gender, hypertension, diabetes mellitus, dyslipidemia, coronary artery disease, SEER summary stage, radiation therapy, and number of cycles of doxorubicin were adjusted in the Cox proportional hazards model, while smoking and BMI were also added to independent variables for analysis. Analyses were performed with gender removed and trastuzumab after doxorubicin added for breast cancer, SEER summary stage removed for hematologic malignancy, and gender removed for gynecologic malignancy. We analyzed survival rates, 95% confidence intervals (CIs), and adjusted HRs (aHRs) of HF in breast cancer, hematologic malignancy, gynecologic malignancy, and sarcoma. All data were analyzed using SAS Windows Ver. 9.4 (SAS Institute Inc., Cary, NC, USA), with *P* < 0.05 considered significant.

## 3 RESULTS

Table 1 shows the baseline characteristics of cancer patients who received chemotherapy including doxorubicin. Of the 58 541 patients, 2324 (4%) were diagnosed with doxorubicin‐induced HF. Compared with patients who did not develop doxorubicin‐induced HF, patients with HF were older and had more advanced stage and comorbidities. Among the oncologic diseases, breast cancer was the most common, followed by non‐Hodgkin lymphoma. The mean number of cycles of doxorubicin was eight, and the proportion of more than four cycles of doxorubicin was higher in cancer patients with doxorubicin‐induced HF.

**Table 1:** Baseline characteristics of cancer patients who received chemotherapy including doxorubicin

Variable	Total	Heart failure (+)	Heart failure (−)	*P*‐value
	(n = 58 541)	(n = 2324) (4%)	(n = 56 217) (96.0%)	
Age (y)	49 ± 12	54 ± 13	49 ± 11	<0.001
Female, n (%)	50 591 (86.4)	1877 (80.8)	48 714 (86.7)	<0.001
Body mass index ≥25 kg/m^2^, n (%)^a^	9574/32 840 (29.2)	449/1335 (33.6)	9125/31 430 (29.0)	<0.001
Smoking (current or former), n (%)^a^	2359/34 109 (6.9)	104/1231 (8.4)	2255/32878 (6.9)	0.010
Hypertension, n (%)	10 747 (18.4)	861 (37.0)	9886 (17.6)	<0.001
Diabetes mellitus, n (%)	6507 (11.1)	423 (18.2)	6084 (10.8)	<0.001
Dyslipidemia, n (%)	4792 (8.2)	256 (11.0)	4536 (8.1)	<0.001
Coronary artery disease, n (%)	1827 (3.1)	228 (9.8)	1599 (2.8)	<0.001
Stage, n (%)^a^				<0.001
Localized	16 843/43 079 (39.1)	480/1524 (31.5)	16 363/41 555 (39.4)	
Regional	20 260/43 079 (47.0)	725/1524 (47.6)	19 535/41 555 (47.0)	
Distant	5976/43 079 (13.9)	319/1524 (20.9)	5657/41 555 (13.6)	
Radiation therapy, n (%)	35 446 (60.5)	1298 (55.9)	34 148 (60.7)	<0.001
Oncological disease, n (%)				<0.001
Breast cancer	43 586 (74.5)	1482 (63.8)	42 104 (74.9)	
Hematologic malignancy				
Non‐Hodgkin lymphoma	10 077 (17.2)	643 (27.7)	9434 (16.8)	
Hodgkin lymphoma	1102 (1.9)	50 (2.2)	1052 (1.9)	
Acute lymphoblastic leukemia	534 (0.9)	31 (1.3)	503 (0.9)	
Acute myeloid leukemia	55 (0.1)	1 (0.0)	54 (0.1)	
Gynecologic malignancy				
Endometrial cancer	985 (1.7)	38 (1.6)	947 (1.7)	
Ovarian cancer	555 (0.9)	18 (0.8)	537 (1.0)	
Uterine sarcoma	60 (0.1)	1 (0.0)	59 (0.1)	
Sarcoma				
Soft tissue sarcoma	971 (1.7)	40 (1.7)	931 (1.7)	
Bone sarcoma	616 (1.1)	20 (0.9)	596 (1.1)	
Doxorubicin (number of cycles)	8 ± 4	9 ± 5	8 ± 4	0.335
1‐4	34 246 (58.5)	1306 (56.2)	32940 (58.6)	0.022
5‐7	19 047 (32.5)	779 (33.5)	18 268 (32.5)	
≥8	5248 (9.0)	239 (10.3)	5009 (8.9)	

Data are presented as mean ± standard deviation.

a Body mass index, smoking, and stage include missing values.

### 3.1 Comparison of variables related to doxorubicin‐induced HF

Of the 43 586 patients with breast cancer, 1482 (3.4%) were diagnosed with doxorubicin‐induced HF. Compared with breast cancer patients who did not develop HF, patients with HF were older and had more advanced stage and many comorbidities. Among the 11 768 patients with hematologic malignancy, 725 (6.2%) were diagnosed with doxorubicin‐induced HF. Compared with patients who did not develop HF, they were older and had more comorbidities (Table 2). In patients with sarcoma, the incidence of doxorubicin‐induced HF was 3.8%. HF patients were older and had lower proportion of female, a higher proportion of hypertension, dyslipidemia, and coronary artery disease. In patients with gynecologic malignancy, the incidence of doxorubicin‐induced HF was 6.6%, and the proportion of hypertension and advanced stage was higher in HF patients (Table S1).

**Table 2:** Comparison of variables related to doxorubicin‐induced heart failure in patients with breast cancer and hematologic malignancy

Variable	Breast cancer			Hematologic malignancy		
	Heart failure (+)	Heart failure (−)	*P*‐value	Heart failure (+)	Heart failure (−)	*P‐*value
	(n = 1482) (3.4%)	(n = 42 104) (96.6%)		(n = 725) (6.2%)	(n = 11 043) (93.8%)	
Age (y)	51 ± 10	47 ± 9	<0.001	61 ± 14	53 ± 16	<0.001
Female, n (%)	1482 (100)	42 104 (100)	1.000	316 (43.6)	4478 (40.6)	0.107
Body mass index ≥25 kg/m^2^, n (%)^a^	279/856 (32.3)	6806/23 949 (28.4)	0.014	150/406 (36.9)	1837/5869 (31.3)	0.018
Smoking (current or former), n (%)^a^	33/799 (4.1)	724/25 183 (2.9)	0.002	62/378 (16.4)	1350/6181 (21.9)	0.516
Hypertension, n (%)	515 (34.8)	6663 (15.8)	<0.001	302 (41.7)	2581 (23.4)	<0.001
Diabetes mellitus, n (%)	229 (15.5)	3885 (9.2)	<0.001	173 (23.9)	1835 (16.6)	<0.001
Dyslipidemia, n (%)	166 (11.2)	3414 (8.1)	<0.001	74 (10.2)	896 (8.1)	0.047
Coronary artery disease, n (%)	111 (7.5)	995 (2.4)	<0.001	104 (14.3)	490 (4.4)	<0.001
Stage, n (%)^a^			<0.001	‐	‐	‐
Localized	302/1012 (29.8)	12 689/32 085 (39.6)				
Regional	626/1012 (61.9)	17 659/32 085 (55.0)				
Distant	84/1012 (8.3)	1737/32 085 (5.4)				
Radiation therapy, n (%)	1056 (71.3)	29 642 (70.4)	0.479	180 (24.8)	3053 (27.6)	0.100
Doxorubicin (number of cycles)	8 ± 5	7 ± 4	0.071	10 ± 5	9 ± 5	0.527
1‐4	859 (58.0)	25 171 (59.8)	0.022	387 (53.4)	6107 (55.3)	0.335
5‐7	467 (31.5)	13 567 (32.2)		262 (36.2)	3704 (33.6)	
≥8	156 (10.5)	3366 (8.0)		76 (10.4)	1232 (11.1)	
Trastuzumab after doxorubicin, n (%)	318 (21.5)	4149 (9.9)	<0.001	‐	‐	‐
Duration of trastuzumab (wk)	41 ± 32	43 ± 30	0.259	‐	‐	‐

Data are presented as mean ± standard deviation.

a Body mass index, smoking, and stage include missing values.

### 3.2 Predictive risk factors for doxorubicin‐induced HF

When adjustments were made except for BMI and smoking due to missing data, the following were predictive risk factors for doxorubicin‐induced HF in patients with breast cancer: age >65 years [aHR 1.34 (1.05‐1.72)], hypertension [aHR 2.45 (2.12‐2.84)], diabetes mellitus [aHR 1.26 (1.05‐1.51)], coronary artery disease [aHR 2.08 (1.63‐2.66)], advanced stage [aHR 1.31 (1.13‐1.50)], and additional trastuzumab administration [aHR 2.94 (2.54‐3.40)]. In patients with hematologic malignancy, the predictive risk factors were age >65 years [aHR 1.75 (1.49‐2.07)], hypertension [aHR 1.62 (1.37‐1.92)], and coronary artery disease [aHR 2.28 (1.80‐2.89)] (Table 3). In sarcoma patients, predictive risk factors were hypertension [aHR 2.52 (1.10‐5.77)] and radiation therapy [aHR 3.29 (1.32‐8.19)], while in patients with gynecologic malignancy, advanced stage [aHR 2.24 (1.04‐4.84)] was a predictive risk factor (Table S2).

**Table 3:** Risk factors for doxorubicin‐induced heart failure in patients with breast cancer and hematologic malignancy

	Breast cancer			Hematologic malignancy		
	aHR	95% CI	*P*‐value	aHR	95% CI	*P*‐value
Age > 65 y	1.34	1.05‐1.72	0.018	1.75	1.49‐2.07	<0.001
Female	‐	‐	‐	0.94	0.81‐1.10	0.467
Hypertension	2.45	2.12‐2.84	<0.001	1.62	1.37‐1.92	<0.001
Diabetes mellitus	1.26	1.05‐1.51	0.014	1.21	1.00‐1.45	0.049
Dyslipidemia	1.09	0.90‐1.33	0.358	1.01	0.78‐1.31	0.930
Coronary artery disease	2.08	1.63‐2.66	<0.001	2.28	1.80‐2.89	<0.001
Advanced stage	1.31	1.13‐1.50	<0.001	‐	‐	‐
Radiation therapy	1.03	0.89‐1.18	0.725	0.94	0.79‐1.12	0.477
Doxorubicin (number of cycles)						
1‐4 (reference)	‐	‐	‐	‐	‐	‐
5‐7	0.99	0.86‐1.14	0.915	1.07	0.90‐1.26	0.452
≥8	1.23	0.99‐1.52	0.063	1.02	0.79‐1.32	0.865
Trastuzumab after doxorubicin	2.94	2.54‐3.40	<0.001	‐	‐	‐

aHR, adjusted hazard ratio; CI, confidence interval.

When adjustments were made including BMI and smoking, the following were predictive risk factors for doxorubicin‐induced HF in patients with breast cancer: hypertension [aHR 2.59 (2.14‐3.13)], coronary artery disease [aHR 2.39 (1.74‐3.28)], and additional trastuzumab administration [aHR 3.15 (2.59‐3.83)]. For patients with hematologic malignancy, the predictive risk factors were age >65 years [aHR 1.84 (1.47‐2.31)], BMI ≥25 kg/m^2^ [aHR 1.28 (1.03‐1.59)], hypertension [aHR 1.49 (1.18‐1.89)], and coronary artery disease [aHR 2.34 (1.69‐3.23)] (Table S3). Hypertension [aHR 3.57 (1.04‐12.26)] was the only predictive risk factor for doxorubicin‐induced HF in patients with sarcoma, and there was no predictive factor for patients with gynecologic malignancy (Table S3).

### 3.3 Survival rates of doxorubicin‐induced HF

**Figure 2:**
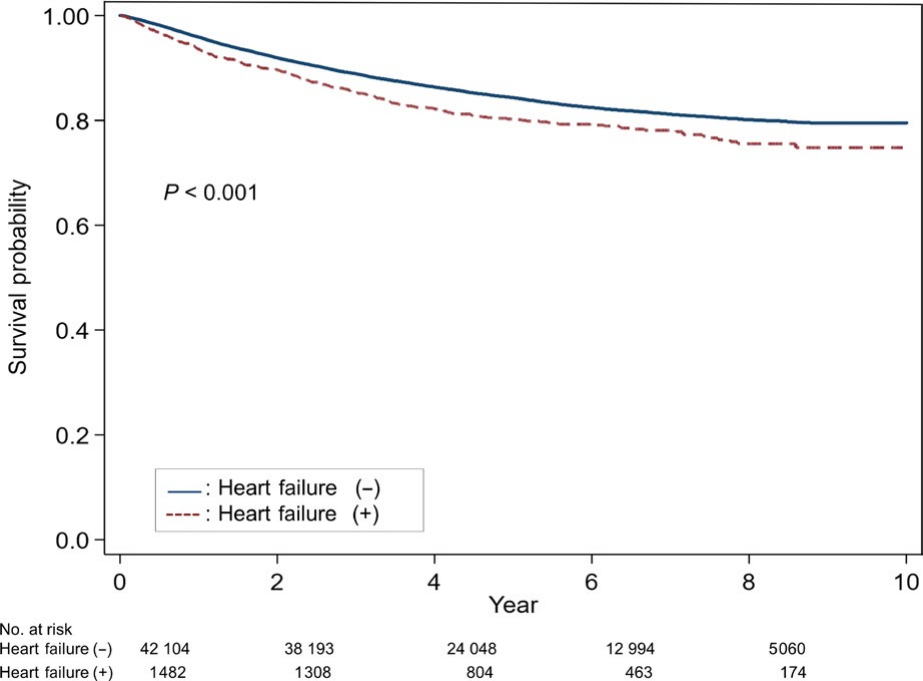
Survival curves of patients with breast cancer who received doxorubicin

As shown in Figure 2, the 5‐year survival rate of breast cancer patients with doxorubicin‐induced HF was 80% while that of breast cancer patients without doxorubicin‐induced HF was 84%. The 8‐year survival rate was 76% in breast cancer patients with doxorubicin‐induced HF and 80% in breast cancer patients without doxorubicin‐induced HF (*P* < 0.001).

**Figure 3:**
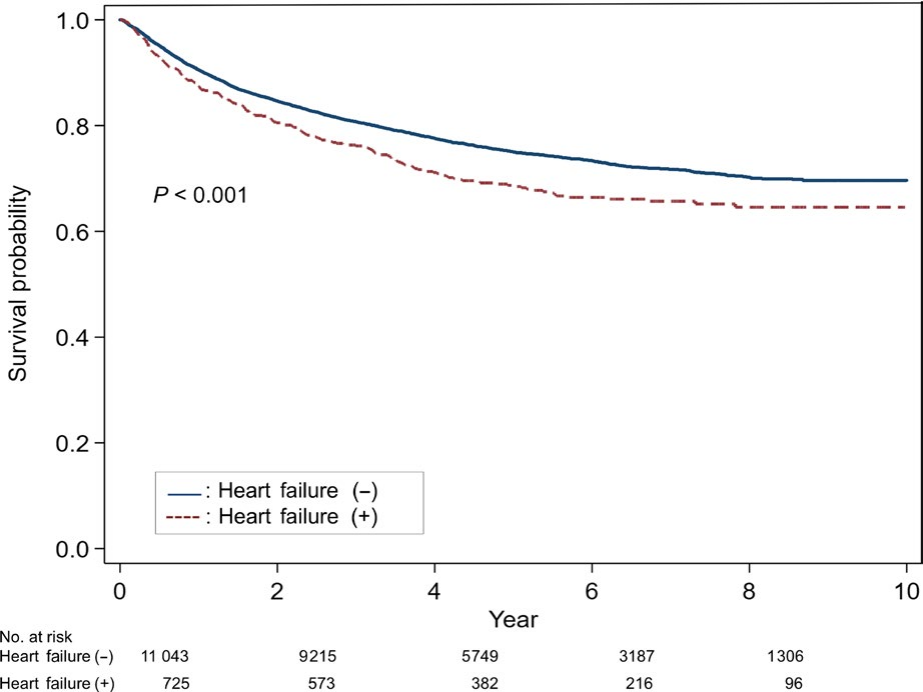
Survival curves of patients with hematologic malignancy who received doxorubicin

In patients with hematologic malignancy, the 5‐year survival rate of patients with doxorubicin‐induced HF was 69% while that of hematologic malignancy patients without doxorubicin‐induced HF was 75%. The 8‐year survival rate was 65% in breast cancer patients with doxorubicin‐induced HF and 70% in breast cancer patients without doxorubicin‐induced HF (*P *< 0.001) (Figure 3).

Finally, in sarcoma patients, the 5‐year survival rate of patients with doxorubicin‐induced HF was 73% while that of patients without doxorubicin‐induced HF was 80% (*P *= 0.138) (Figure S1).

## 4 DISCUSSION

We showed that the incidence of doxorubicin‐induced HF in Korean cancer patients was about 4%. In patients with breast cancer, predictive risk factors for doxorubicin‐induced HF were old age, hypertension, coronary artery disease, advanced stage, and additional trastuzumab administration. Similarly, for hematologic malignancy, predictive risk factors were old age, hypertension, and coronary artery disease. Furthermore, our data revealed doxorubicin‐induced HF significantly lowered 5‐year survival in patients with breast cancer and hematologic malignancy compared with non‐HF patients.

In our study, we defined HF according to diagnostic codes. Generally, HF can be classified as stage A (at high risk), B (asymptomatic), C (symptomatic), and D (refractory).^16^ Patients treated with doxorubicin are automatically classified as stage A. Our cohort mainly consisted of patients with stage C HF, where HF incidence closely related to the accumulated dose of doxorubicin. A previous study reported that the incidence of doxorubicin‐induced HF was 2.2% and the cumulative probability of congestive HF relative to the total cumulative dose of doxorubicin was 3% at 400 mg/m^2^, 7% at 550 mg/m^2^, and 18% at 700 mg/m^2^.^17^ However, patients with stage B HF were not included in this study, and consequently, the incidence of HF may have been underestimated. Swain et al^12^ found that when HF was defined based on clinical signs and symptoms, the incidence of doxorubicin‐induced HF was 5.1% and the estimated cumulative percentage of congestive HF relative to the total cumulative dose of doxorubicin was 5% at 400 mg/m^2^, 26% at 550 mg/m^2^, and 48% at 700 mg/m^2^. These authors also observed that the incidence of cardiac events, defined by the development of congestive HF and cardiac dysfunction, was 23.7%. Relative to the total cumulative dose of doxorubicin, the incidence of cardiac events was 9% at 250 mg/m^2^, 32% at 400 mg/m^2^, 65% at 550 mg/m^2^, and 86% at 700 mg/m^2^.^12^ Thus when stage B HF is included, the incidence of doxorubicin‐induced HF increases from twofold to sixfold depending on the cumulative dose of doxorubicin. In a separate study that defined cardiotoxicity as LV ejection fraction (LVEF) decrease >10 absolute points and final LVEF <50% and included stages B and C HF, the incidence of cardiotoxicity was 9% under a mean cumulative anthracycline dose of 360 mg/m^2^.^18^

Previous studies indicate that risk factors for doxorubicin‐induced HF include old age (>65 years), female, preexisting cardiac disease, hypertension, accumulated dose, mediastinal radiation therapy, and concomitant chemotherapy (alkylating agents or trastuzumab).^13^, ^19^, ^20^, ^21^, ^22^ However, these were risk factors for Western cancer patients. In Korea, doxorubicin‐induced HF was more likely to occur in female cancers such as breast cancer and gynecologic malignancy. However, the female gender was not a risk factor for doxorubicin‐induced HF in Korean hematologic malignancy and sarcoma patients. In addition, cumulative dose estimated as the number of cycles of doxorubicin, and mediastinal radiation were not risk factors for doxorubicin‐induced HF, while old age (>65 years), hypertension, coronary artery disease, and additional trastuzumab administration were significant risk factors. Although being overweight (BMI 25‐29.9 kg/m^2^) or obese (BMI ≥30 kg/m^2^) is previously reported risk factors for anthracycline‐induced cardiotoxicity,^23^ we could not accurately confirm this in our study due to missing BMI data. Previous reports indicate that the most consistent risk factor for cardiotoxicity associated with doxorubicin and/or trastuzumab is hypertension.^24^ In our study, hypertension was the only common risk factor for predicting of doxorubicin‐induced HF among patients with breast cancer, hematologic malignancy, and sarcoma.

The 5‐year survival rate stratified by HF stage is 97% for stage A, 96% for stage B, 75% for stage C, and 20% for stage D.^25^ Overall, the 5‐year survival rate of HF is approximately 50%, which is worse than that of breast cancer, colon cancer, and lymphoma.^2^ Patients with doxorubicin‐induced HF have an especially poor prognosis. Felker et al^14^ reported the median survival of doxorubicin‐induced cardiomyopathy is about one year, and the prognosis is worse than that of ischemic cardiomyopathy. However, in this study, only 15 of 1230 patients (1%) had doxorubicin‐induced HF, which is a limitation compared to HF due to other causes. We compared our data with the Korean Heart Failure (KorHF) registry to analyze survival rates between doxorubicin‐induced HF and HF due to other causes. The KorHF registry was a nationwide, prospective, observational, multicenter registry that included 3200 patients with acute HF from 2004 to 2009. The underlying causes were mainly ischemic heart disease (52.3%), idiopathic cardiomyopathy (23.3%), and valvular heart disease (12.7%).^26^ In the KorHF registry, the survival rates of patients with HF at 1, 2, 3, and 4 years were 85%, 79%, 74%, and 70%, respectively. The 5‐year survival rate was 61% for male and 69% for female.^26^ Alternatively, in our study, the survival rates of doxorubicin‐induced HF in breast cancer patients at 1, 2, 3, 4, and 5 years were 94%, 90%, 85%, 82%, and 80%, respectively. In addition, the survival rates of doxorubicin‐induced HF in hematologic malignancy patients at 1, 2, 3, 4, and 5 years were 87%, 81%, 76%, 71%, and 69%, respectively. Therefore, the survival rate of doxorubicin‐induced HF in Koreans is significantly better than the previous result, in which the survival rate of doxorubicin‐induced cardiomyopathy was <50% reported by Felker et al^14^ and the prognosis of doxorubicin‐induced HF in Koreans was not worse compared with other causes of HF. Furthermore, cancer patients with doxorubicin‐induced HF have a lower survival rate than those without HF. As shown in Figures 2 and 3, the 5‐year survival rates of breast cancer and hematologic malignancy patients with doxorubicin‐induced HF were 80% and 69%, respectively, compared with the 5‐year survival rates of 84% and 75% of breast cancer and hematologic malignancy patients without HF. Because this difference in survival rates continues to be observed after 5 years, doxorubicin‐induced HF has an effect on the mortality rate of cancer survivors. Doxorubicin‐induced HF also reduces quality of life by causing dyspnea, fatigue, and related symptoms in cancer survivors.^3^, ^27^ Therefore, prevention and early diagnosis and treatment of doxorubicin‐induced HF can both increase patient survival and improve quality of life for cancer survivors.

Our study has several limitations. First, there is a lack of data on echocardiographic findings (esp, LVEF) or biomarkers. Because our study was based on NHIS, it was difficult to know the detailed test results and of individual patients. Second, because we primarily included patients with stage C (symptomatic) HF, we presume that stage B (asymptomatic) HF was excluded from this study. According to the Multi‐Ethnic Study of Atherosclerosis, 18.6% of participants with asymptomatic HF developed overt HF within 9 years.^28^ Therefore, our reported incidence of HF may be underestimated given that the number of patients with stage B HF is over three times higher than those with stage C HF.^25^ Third, we could not confirm the effect of other chemotherapeutics such as cyclophosphamide, which is frequently used in combination therapy with doxorubicin to induce cardiomyopathy. Fourth, we could not accurately confirm the cumulative dose of doxorubicin for each patient because doxorubicin regimens vary among different types of cancer: 4‐6 cycles of 50‐60 mg/m^2 ^for breast cancer, 6‐8 cycles of 40‐50 mg/m^2 ^for lymphoma, and 6‐8 cycles of 75‐90 mg/m^2^ for sarcoma.^29^ Therefore, we estimated cumulative dosage by the number of cycles of doxorubicin. According to the mathematical model predicting the likelihood of doxorubicin‐induced HF, that is, *y* = *x* ^2^/16 where *x* is the number of cycles of 50 mg/m^2^ each and *y* is the percent of prevalence of doxorubicin‐induced HF, the incidence of HF is 4% at eight cycles.^30^ This was consistent with data from our cohort. Despite these limitations, our study still identified significant risk factors that predicted doxorubicin‐induced HF in cancer patients and assessed the effect of doxorubicin‐induced HF on cancer survival in a nationwide cohort. Because the data from Asian population in terms of cardio‐oncology are scarce, our study also tried to fill that gap.

In conclusion, management of risk factors, early detection, and treatment for doxorubicin‐induced HF in cancer patients treated with chemotherapy including doxorubicin might be critical for patient survival.

## CONFLICT OF INTEREST

The authors declare no conflict of interests.
