# Supplementary figures and images for: Doxorubicin‐induced heart failure in cancer patients: A cohort study based on the Korean National Health Insurance Database
Source: Cancer Med. 2018 Nov 19;7(12):6084–92. doi: 10.1002/cam4.1886 (PMC6308087; doi:10.1002/cam4.1886)

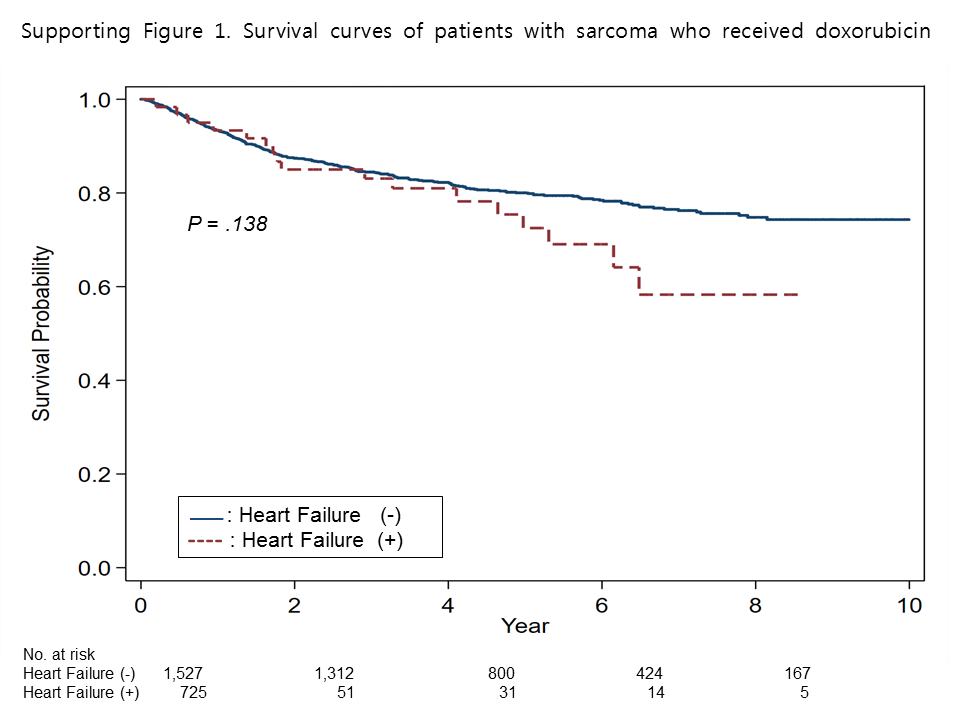

Supplement: Supplementary file 1 [file CAM4-7-6084-s001.tif]
