# Supplementary material for: Doxorubicin‐induced heart failure in cancer patients: A cohort study based on the Korean National Health Insurance Database
Source: Cancer Med. 2018 Nov 19;7(12):6084–92. doi: 10.1002/cam4.1886 (PMC6308087; doi:10.1002/cam4.1886)
Supplement: Supplementary file 2 [file CAM4-7-6084-s002.docx]

Supporting Table 1. Comparison of variables related to doxorubicin-induced heart failure in patients with sarcoma and gynecologic malignancy

| Characteristic | Sarcoma | | | Gynecologic malignancy | | |
| --- | --- | --- | --- | --- | --- | --- |
|  | Heart Failure (+) | Heart Failure (-) | p-value | Heart Failure (+) | Heart Failure (-) | p-value |
|  | (n = 60) (3.8%) | (n = 1,527) (96.6%) |  | (n = 57) (6.6%) | (n = 1,543) (96.4%) |  |
| Age (years) | 49 ± 15 | 43 ± 16 | 0.005 | 55 ± 10 | 53 ± 10 | 0.108 |
| Female, n (%) | 22 (36.7) | 589 (38.6) | 0.766 | - | - | - |
| Body mass index ≥ 25 kg/m^2^, n (%)^*^ | 12/32 (37.5) | 207/732 (28.3) | 0.259 | 8/32 (25.0) | 275/880 (31.3) | 0.453 |
| Smoking (current or former), n (%)^*^ | 9/29 (31.0) | 162/668 (24.2) | 0.937 | 0/25 (0.0) | 19/846 (2.2) | 0.267 |
| Hypertension, n (%) | 23 (38.3) | 262 (17.2) | <0.001 | 21 (36.8) | 380 (24.6) | 0.037 |
| Diabetes mellitus, n (%) | 11 (18.3) | 173 (11.3) | 0.097 | 10 (17.5) | 191 (12.4) | 0.248 |
| Dyslipidemia, n (%) | 7 (11.7) | 69 (4.5) | 0.011 | 9 (15.8) | 157 (10.2) | 0.172 |
| Coronary artery disease, n (%) | 8 (13.3) | 52 (3.4) | <0.001 | 5 (8.8) | 62 (4.0) | 0.079 |
| Stage, n (%)^*^ |  |  | 0.851 |  |  | 0.022 |
| Localized | 18/32 (56.3) | 553/934 (59.2) |  | 9/46 (19.6) | 359/1,130 (31.8) |  |
| Regional | 6/32 (18.8) | 141/934 (15.1) |  | 11/46 (23.9) | 358/1,130 (31.7) |  |
| Distant | 8/32 (25.0) | 240/934 (25.7) |  | 26/46 (56.5) | 413/1,130 (36.5) |  |
| Radiation therapy, n (%) | 36 (60.0) | 761 (49.8) | 0.123 | 26 (45.6) | 692 (44.8) | 0.909 |
| Doxorubicin (number of cycles) | 10 ± 7 | 11 ± 7 | 0.007 | 8 ± 6 | 8 ± 5 | 0.966 |
| 1-4 | 30 (50.0) | 764 (50.0) | 0.045 | 30 (52.6) | 898 (58.2) | 0.260 |
| 5-7 | 26 (43.3) | 493 (32.3) |  | 24 (42.1) | 504 (32.7) |  |
| ≥8 | 4 (6.7) | 270 (17.7) |  | 3 (5.3) | 141 (9.1) |  |

Data are presented as mean ± standard deviation.

^*^ Body mass index, smoking, and stage include missing values.

Supporting Table 2. Risk factors of doxorubicin-induced heart failure in patients with sarcoma and gynecologic malignancy

|  | Sarcoma | | | Gynecologic malignancy | | |
| --- | --- | --- | --- | --- | --- | --- |
|  | aHR | 95% CI | p-value | aHR | 95% CI | p-value |
| Age > 65 years | 0.42 | 0.10-1.85 | 0.252 | 1.20 | 0.53-2.73 | 0.659 |
| Female | 1.43 | 0.62-3.30 | 0.400 | - | - | - |
| Hypertension | 2.52 | 1.10-5.77 | 0.029 | 1.68 | 0.88-3.21 | 0.118 |
| Diabetes mellitus | 0.98 | 0.35-2.76 | 0.975 | 1.30 | 0.60-2.82 | 0.503 |
| Dyslipidemia | 1.97 | 0.57-6.81 | 0.284 | 1.44 | 0.64-3.24 | 0.382 |
| Coronary artery disease | 0.64 | 0.08-4.95 | 0.671 | 1.94 | 0.73-5.12 | 0.182 |
| Advanced stage | 1.18 | 0.55-2.54 | 0.668 | 2.24 | 1.04-4.84 | 0.040 |
| Radiation therapy | 3.29 | 1.32-8.19 | 0.010 | 0.92 | 0.51-1.68 | 0.792 |
| Doxorubicin (number of cycles) |  |  |  |  |  |  |
| 1-4 (reference) | - | - | - | - | - | - |
| 5-7 | 1.21 | 0.54-2.67 | 0.644 | 1.40 | 0.76-2.58 | 0.275 |
| ≥8 | 0.43 | 0.10-1.91 | 0.268 | 0.57 | 0.13-2.45 | 0.454 |

aHR, adjusted hazard ratio; CI, confidence interval.

Supporting Table 3. Risk factors of doxorubicin-induced heart failure including BMI, smoking

|  | Breast cancer | | | Hematologic malignancy | | | Sarcoma | | | Gynecologic malignancy | | |
| --- | --- | --- | --- | --- | --- | --- | --- | --- | --- | --- | --- | --- |
|  | aHR | 95% CI | p | aHR | 95% CI | p | aHR | 95% CI | p | aHR | 95% CI | p |
| Age > 65 years | 1.12 | 0.78-1.61 | 0.536 | 1.84 | 1.47-2.31 | <0.001 | 0.00 | 0.00-. | 0.992 | 0.66 | 0.14-3.05 | 0.594 |
| Female | - | - | - | 0.98 | 0.79-1.21 | 0.818 | 1.34 | 0.40-4.49 | 0.636 | - | - | - |
| BMI ≥ 25 (kg/m^2^) | 1.08 | 0.90-1.29 | 0.414 | 1.28 | 1.03-1.59 | 0.028 | 0.97 | 0.29-3.22 | 0.958 | 0.60 | 0.22-1.65 | 0.324 |
| Smoking | 1.24 | 0.96-1.60 | 0.105 | 1.12 | 0.84-1.49 | 0.459 | 0.28 | 0.03-2.70 | 0.271 | 0.36 | 0.05-2.75 | 0.325 |
| Hypertension | 2.59 | 2.14-3.13 | <0.001 | 1.49 | 1.18-1.89 | 0.001 | 3.57 | 1.04-12.26 | 0.043 | 1.74 | 0.68-4.42 | 0.248 |
| Diabetes mellitus | 1.26 | 0.99-1.61 | 0.056 | 1.20 | 0.94-1.55 | 0.150 | 0.88 | 0.20-3.82 | 0.867 | 2.11 | 0.77-5.75 | 0.145 |
| Dyslipidemia | 1.10 | 0.85-1.41 | 0.466 | 1.09 | 0.78-1.52 | 0.634 | 1.61 | 0.20-13.29 | 0.659 | 1.42 | 0.44-4.55 | 0.560 |
| Coronary artery disease | 2.39 | 1.74-3.28 | <0.001 | 2.34 | 1.69-3.23 | <0.001 | 1.35 | 0.15-12.41 | 0.789 | 1.36 | 0.29-6.31 | 0.694 |
| Advanced stage | 1.16 | 0.97-1.39 | 0.112 | - | - | - | 1.01 | 0.32-3.17 | 0.992 | 1.93 | 0.71-5.28 | 0.201 |
| Radiation therapy | 1.06 | 0.86-1.29 | 0.520 | 1.02 | 0.80-1.28 | 0.903 | 1.96 | 0.59-6.45 | 0.271 | 1.20 | 0.51-2.79 | 0.679 |
| Doxorubicin  (number of cycles) |  |  |  |  |  |  |  |  |  |  |  |  |
| 1-4 (reference) | - | - | - | - | - | - | - | - | - | - | - | - |
| 5-7 | 0.96 | 0.79-1.15 | 0.636 | 1.09 | 0.87-1.37 | 0.437 | 1.50 | 0.45-5.00 | 0.512 | 1.05 | 0.43-2.57 | 0.918 |
| ≥8 | 1.24 | 0.91-1.68 | 0.171 | 0.96 | 0.66-1.39 | 0.816 | 2.28 | 0.41-12.71 | 0.347 | 0.86 | 0.11-6.77 | 0.883 |
| Trastuzumab | 3.15 | 2.59-3.83 | <0.001 | - | - | - | - | - | - | - | - | - |

aHR, adjusted hazard ratio; BMI, body mass index; CI, confidence interval.
